# Shifting Paradigms in Antifungal Prophylaxis and Their Effects on Candidemia Outcomes in Hematological Malignancies: A 14-Year Experience from a Single Tertiary Center

**DOI:** 10.3390/jof11090630

**Published:** 2025-08-28

**Authors:** Fazıl Çağrı Hunutlu, Fahir Özkalemkaş, Beyza Ener, Dilay Demirayak, Büşra Çalışır, Hikmet Öztop, İbrahim Ethem Pınar, Vildan Gürsoy, Tuba Ersal, Tuba Güllü Koca, Emin Halis Akalın, Vildan Özkocaman

**Affiliations:** 1 Division of Hematology, Department of Internal Medicine, Faculty of Medicine, Bursa Uludag University, Gorukle Campus, Bursa 16059, Turkey; fahir@uludag.edu.tr (F.Ö.); dr.ethem@hotmail.com (İ.E.P.); vildanterzioglu@hotmail.com (V.G.); tubaersal@uludag.edu.tr (T.E.); vildanoz@uludag.edu.tr (V.Ö.); 2 Department of Microbiology, Faculty of Medicine, Bursa Uludag University, Bursa 16059, Turkey; bener@uludag.edu.tr (B.E.); busraozeycalisir@gmail.com (B.Ç.); 3 Department of Gastroenterology, Bilecik Education and Research Hospital, Bilecik 11000, Turkey; dilaydemirayak@gmail.com; 4 Department of Internal Medicine, Faculty of Medicine, Bursa Uludag University, Bursa 16059, Turkey; hikmetoztop@uludag.edu.tr; 5 Department of Hematology, Bursa City Hospital, Bursa 16059, Turkey; tubakocamd@gmail.com; 6 Department of Infectious Diseases and Clinical Microbiology, Faculty of Medicine, Bursa Uludag University, Bursa 16059, Turkey; halis@uludag.edu.tr

**Keywords:** candidemia, antifungal prophylaxis, posaconazole, hematological malignancies, acute myeloid leukemia, *Candida tropicalis*

## Abstract

Evolving antifungal prophylaxis approaches have reshaped candidemia patterns and outcomes in hematological malignancy (HM) patients. This study aimed to evaluate temporal changes in candidemia incidence, species distribution, and factors associated with mortality in relation to prophylaxis practices. Adult HM patients with candidemia between 2009 and 2023 were included. Clinical and microbiological data were analyzed, and candidemia rates were compared across different prophylaxis periods. Sixty-six patients were identified, with acute myeloid leukemia (AML) being the most common underlying malignancy (40.9%). Non-albicans *Candida* species predominated, especially *C. krusei* and *C. tropicalis*. In AML patients, candidemia incidence significantly decreased over time (β = −0.694, *p* = 0.004), with the lowest rates observed during the extended-release posaconazole tablet era (2016–2023). However, 30-day mortality remained high (53%) and unchanged across periods. Multivariate analysis identified *C. tropicalis* and total parenteral nutrition as independent risk factors for 30-day mortality (OR: 4.3 and 4.6, *p* < 0.05), while antifungal prophylaxis was protective (OR: 0.07, *p* = 0.017). In patients with AML, posaconazole prophylaxis, particularly in the extended-release tablet formulation, significantly reduced the incidence of candidemia. However, overall 30-day mortality rates remained high, with *C. tropicalis* being a major contributor. Thus, individualized prophylaxis and treatment strategies are crucial for improving outcomes.

## 1. Introduction

The incidence of invasive fungal infections has increased worldwide in recent years [1]. Among immunosuppressed patients with hematological malignancies (HMs), fungemias are the most common form of fungal infection, with *Candida* species representing the principal etiological agents. Various studies report different incidence rates of candidemia, and the overall incidence among patients hospitalized due to HMs ranges between 0.5% and 1.5% [2]. Numerous factors influence this incidence, including geographical distribution, the presence and type of antifungal prophylaxis, physical conditions in the hospital, and the characteristics of the underlying hematological malignancy [3,4]. Factors such as broad-spectrum antibiotic and systemic steroid use, the presence of intravenous catheters, total parenteral nutrition (TPN), multiple hospital and intensive care unit admissions, and prolonged neutropenia particularly predispose patients with HMs to developing candidemia [3,5,6].

Advances in antifungal prophylaxis and empirical therapies over the years have resulted in changes in the epidemiology of the development of candidemia in patients with HMs. In particular, infections caused by non-albicans *Candida* (NAC) species have increased, and these species can develop resistance to commonly used antifungal agents. However, the incidence of *C. albicans* has decreased over the years [2,7,8]. Although their incidence varies by geographical region, the most frequently encountered NAC species are *C. krusei*, *C. tropicalis*, *C. parapsilosis species complex and C. glabrata* [9]. Among NAC species, *C. parapsilosis species complex* and *C. tropicalis* in particular have the ability to form biofilms [10,11]. Further, *C. tropicalis* disrupts host tissue cellular integrity through biofilm formation and via the production of extracellular hydrolytic enzymes such as proteinase, phospholipase, and hemolysin [12]. As *C. tropicalis* possesses multiple virulence factors and the potential for development of fluconazole resistance, it has been identified as the strain associated with the highest mortality in some candidemia series [13].

Candidemia cases developing under antifungal prophylaxis are defined as breakthrough candidemia (BrC). Over the years, there have been notable changes in both the frequency of these cases and the distribution of the associated *Candida* species [2]. This shift has become particularly pronounced over time in acute myeloid leukemia (AML) cases with the use of triazoles (fluconazole, itraconazole, voriconazole and posaconazole) [9]. Posaconazole is a triazole with activity against yeasts and molds. The introduction of posaconazole, specifically for prophylaxis in AML cases, has resulted in a decrease in the global frequency of fungal infections over time [14,15]. Posaconazole penetrates biofilm layers effectively and is less affected by resistance mechanisms arising from mutations in the ergosterol gene (ERG11) and efflux pumps compared to other azoles due to its lipophilic structure [8,16,17].

Despite all advancements in prophylaxis strategies and empirical therapy, mortality in candidemia cases can reach up to 60% due to the nonspecific nature of clinical findings [18]. Therefore, delineation of local epidemiological data and resistance profiles at each center may contribute to reducing mortality rates by enabling the initiation of appropriate treatment early in the course of candidemia cases. In this study, we aimed to evaluate the changes in the incidence of candidemia and the causative strains resulting from evolving prophylaxis strategies over the years and to evaluate the factors influencing mortality.

## 2. Materials and Methods

### 2.1. Study Population and Data Collection

This retrospective descriptive cohort study included patients aged 18 years and older hospitalized at the Bursa Uludag University Faculty of Medicine Hematology Clinic due to HMs between January 2009 and December 2023. Patients with *Candida* isolated from blood cultures during hospitalization for HMs were included in the study. Those with *Candida* isolated in body fluids other than blood culture, concurrent solid organ malignancy or HIV positivity, and patients who developed candidemia during hematopoietic stem cell transplantation were excluded.

For each case, we documented the demographic information (age, sex, comorbidities), baseline treatments (previous hospitalizations, prior treatments), as well as the type and history of the disease. We documented several risk factors associated with candidemia, including the use of central venous catheters (CVCs), administration of TPN, utilization of broad-spectrum antibacterial agents, prior administration of systemic steroids, and approaches to antifungal prophylaxis and treatment. Patient data were obtained retrospectively from medical files and the hospital information management system.

### 2.2. Definitions

Candidemia is characterized by the isolation of any *Candida* species from a single blood culture set in a patient exhibiting signs and symptoms of infection. BrC was defined as an infection occurring in patients receiving systemic antifungal agents with known activity against Candida species for at least 7 days before the onset of candidemia, either for prophylaxis or empiric therapy. De novo candidemia was defined as infection occurring in patients who were not receiving systemic antifungal therapy at the time of the candidemia [19]. Corticosteroid exposure was defined as receipt of prednisone at a dose of at least 20 mg or its equivalent within the 10 days preceding the onset of candidemia. Broad-spectrum antibiotics were defined as piperacillin/tazobactam, third- or fourth-generation cephalosporins, or carbapenems. Catheter-related candidemia was defined when the time to positivity for a *Candida* species from the CVC blood culture was more than 2 h faster than that from a simultaneously drawn peripheral blood culture. The decision to remove the CVC was made on a case-by-case basis, taking into account factors such as the presumed source of candidemia, bleeding risk, and vascular access availability. Neutropenia was defined as an absolute neutrophil count (ANC) <500 cells/mm^3^, or an ANC expected to decrease to this level within 48 h based on serial measurements; severe neutropenia was defined as an ANC <100 cells/mm^3^ [20].

During the study period, standard protocols for antifungal prophylaxis consisted of oral fluconazole tablets in patients with acute lymphoblastic leukemia (ALL) undergoing remission-induction, consolidation, or salvage chemotherapy, as well as in non-Hodgkin lymphoma (NHL) and Hodgkin lymphoma (HL) patients receiving salvage regimens. In patients with multiple myeloma, routine antifungal prophylaxis was not administered outside of autologous stem cell transplantation. The primary antifungal prophylaxis used in AML patients hospitalized for remission-induction or salvage therapy has changed over the years. While fluconazole tablets were used during 2009–2010, posaconazole suspension was used during 2011–2015, and posaconazole extended-release tablets were used during 2016–2023. For AML consolidation therapy, fluconazole tablets were employed throughout the study period. Doses of antifungal agents used for prophylaxis were determined in accordance with established guidelines [20,21]. In candidemia cases, initial antifungal therapy was defined as the agent initiated upon notification of a positive *Candida* signal in blood cultures. Inadequate clinical response was characterized by persistent candidemia despite appropriate antifungal treatment or ongoing signs of infection and clinical deterioration not attributable to other causes.

The incidence of candidemia in AML cases was calculated using the formula “(Number of candidemia episodes in patients with AML in 1 year/Total hospital-days for AML patients in that year) × 1000, and the incidence of candidemia-related 30-day mortality was calculated using the formula “(Number of candidemia-related deaths (within 30 days) occurring in patients with AML in one year/Total hospital-days for AML patients in that year) × 1000. Candidemia-related 30-day mortality was defined as death from any cause occurring within 30 days of the first positive blood culture for *Candida*.

### 2.3. Microbiology and Susceptibility Tests

Blood specimens were collected from patients with suspected candidemia under sterile conditions. Samples were inoculated into BACTEC™ 9000 automated blood culture bottles (Becton Dickinson, Sparks, MD, USA) and incubated for up to 5 days. Gram staining was performed on samples that showed a positive growth signal during incubation. Samples in which yeast cells were observed on Gram staining were inoculated on Sabouraud dextrose agar (SDA) and 5% sheep blood agar plates containing chloramphenicol and gentamicin. Species were identified using conventional identification methods (Germ-tube test, cornmeal-Tween 80 agar morphology and Chrom Agar *Candida*). API ID 32 C (bioMérieux, Marcy-l’Étoile, France) was used until 2019. From 2019, identification was carried out using matrix-assisted laser desorption/ionization time-of-flight mass spectrometry (MALDI-TOF MS; Bruker^®^, Bremen, Germany). Isolates stored at −80 °C during the study period were revived and tested in vitro for antifungal susceptibility according to Clinical and Laboratory Standards Institute (CLSI) M27 and M60 reference methods [22,23]. Results were interpreted as susceptible (S), intermediate/dose-dependent susceptible (SDD/I) or resistant (R) based on clinical breakpoints. In the absence of clinical breakpoints, epidemiological cutoff values were used to classify isolates as wild type (WT) or non-wild type (NWT) [24]. Quality control was performed using *C. parapsilosis ATCC 22019* and *C. krusei ATCC 6258* reference strains.

### 2.4. Statistical Analysis

Descriptive statistics were presented as frequencies and percentages for categorical variables. For continuous variables, mean ± standard deviation was used for normally distributed data, and median with interquartile range (IQR) was used otherwise, as assessed using the Shapiro–Wilk test. Incidence rates of candidemia were reported as medians with IQR per 1000 patient-days. Temporal distribution of *C. albicans* and NAC species between 2009 and 2023 was visualized using stacked bar plots, whereas annual incidence and mortality trends in AML patients were depicted using line graphs. To assess trends in AML candidemia incidence over time, simple linear regression was applied with year as the independent variable and incidence as the dependent variable. To evaluate the trend in candidemia-related 30-day mortality incidence over time, a simple linear regression analysis was conducted using year as the independent variable and mortality incidence as the dependent variable. Due to non-normal distribution, comparisons among three antifungal prophylaxis periods were performed using the Kruskal–Wallis test. For two-group comparisons, the Mann–Whitney U test was used for continuous variables, and the Chi-square test for categorical variables. For categorical variables with more than two groups, the Fisher–Freeman–Halton exact test was applied where appropriate. Factors associated with 30-day mortality were evaluated using binary logistic regression. Variables with a *p*-value <0.20 in univariate analyses were included in a multivariate model using the backward likelihood ratio (Backward LR) method. Statistical significance was defined as a *p*-value <0.05 in multivariate models. Survival analysis was performed using the Kaplan–Meier method, and survival curves were compared using the log-rank test. All analyses were conducted using IBM SPSS Statistics for Windows, Version 29.0 (IBM Corp., Armonk, NY, USA).

## 3. Results

### 3.1. Characteristics of Patients with Candidemia

During the study period (January 2009–December 2023), 66 patients developed candidemia. Their clinical and demographic characteristics are summarized in Table 1. The median age of the study group was 48.5 years, with a male predominance (57.6%). AML was the most common underlying HMs (40.9%), whereas NHL was the most frequent disease in the non-leukemic group (21.2%). The majority of patients developing candidemia (57.6%) were relapsed/refractory cases, whereas candidemia episodes during consolidation therapy were observed in eight cases. Among predisposing factors, nearly all patients (95.4%) had received broad-spectrum antibiotics, and 36.4% received TPN. BrC was identified as the candidemia type in the vast majority of cases (81.8%), whereas the rate of de novo infection was 18.2%. The 30-day candidemia-related mortality rate for the entire cohort was 53%.

### 3.2. Distribution and Temporal Trends of Candida Species

**Figure 1:**
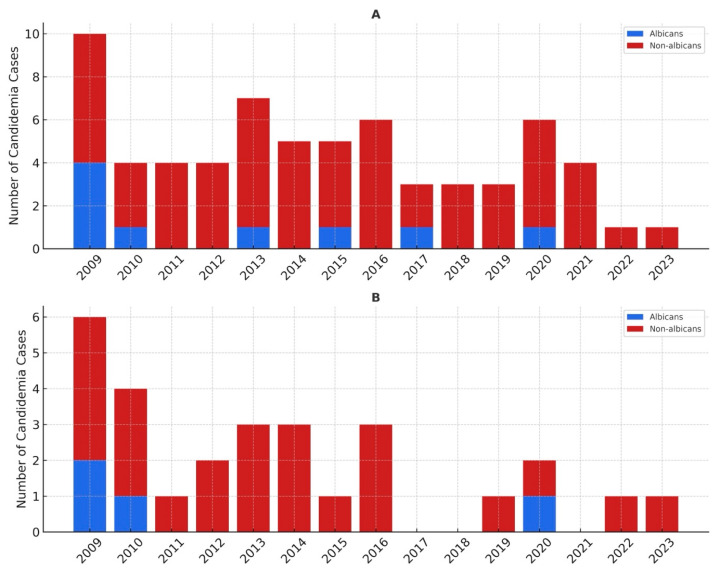
Annual distribution of *Candida* species, (**A**) overall cohort, (**B**) AML patients.

The distribution of *Candida* species in patients with AML and the non-AML group is presented in Table 2. The distribution of causative species between the two groups was not significantly different (*p* > 0.05). The most frequently isolated strains in both groups were NAC species; *C. krusei* was followed by *C. tropicalis*. *C. albicans* was found to be the fourth most common strain. The annual distribution of candidemia cases is presented in Figure 1. The highest number of cases occurred in 2009 (*n* = 10), and NAC species remained the leading causes of candidemia in each year.

**Figure 2:**
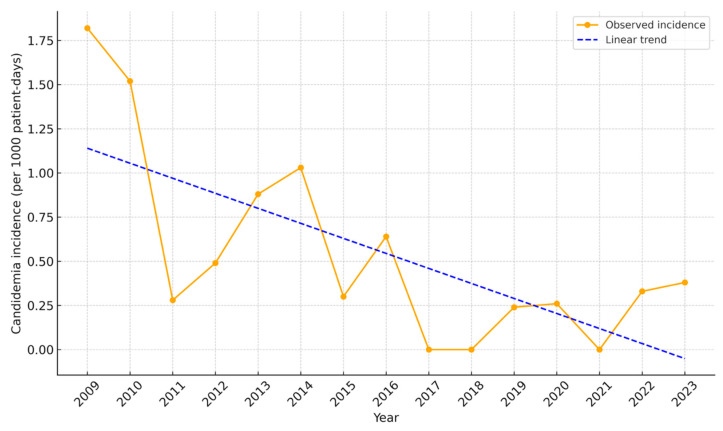
Declining trend in candidemia incidence among AML patients.

Patients with AML were stratified into three groups according to the different prophylaxis strategies used. Candidemia incidence by period, and its temporal changes, are presented in Table 3 and Figure 2. The highest incidence of candidemia was observed before 2011 (1.67 per 1000 hospital-days), with a marked decline thereafter. Linear regression analysis confirmed a statistically significant decrease in candidemia incidence in patients with AML over time (β = –0.694; 95% CI, –0.138 to –0.032; *p* = 0.004; R^2^ = 0.48). Candidemia incidences were compared among three time periods. A statistically significant difference was found between the periods 2009–2010 and 2016–2023 (1.67 vs. 0.27, *p* = 0.022), whereas other inter-period differences were not statistically significant.

**Figure 3:**
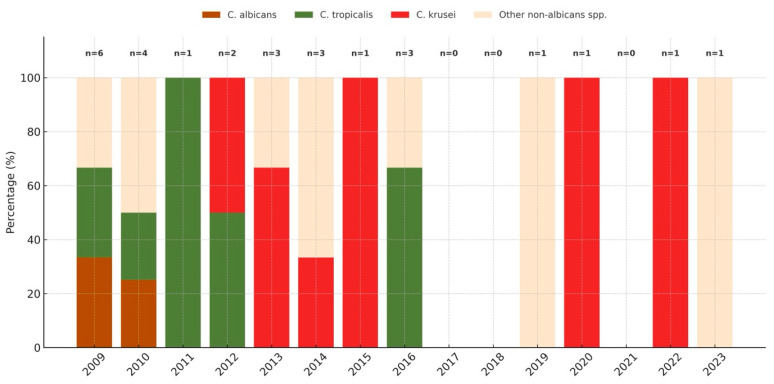
Trends in proportions of *C. albicans* and non-albicans *Candida* species over time in AML-related candidemia cases.

The yearly distribution of *Candida* species in patients with AML is shown in Figure 3. Notably, no *C. albicans* cases were observed after 2011, and no *C. tropicalis* cases were observed after 2016.

### 3.3. Antifungal Therapy and Drug Susceptibility

Antifungal prophylaxis and treatment strategies for candidemia in patients with AML and non-AML hematologic malignancies are outlined in Table 4. Prophylaxis was administered to all patients with AML, compared to 69.2% of the non-AML patients (*p* < 0.001). Fluconazole was the most frequently used prophylactic agent in both groups (66.7% vs. 74.1%). Posaconazole prophylaxis was employed exclusively in patients with AML. Empirically, L-AmB was the most preferred initial therapeutic agent for the treatment of candidemia in both groups (51.9% vs. 51.3%). The rate of echinocandin use as first-line therapy was similar in both groups (18.5% vs. 17.9%). The rate of switching first-line therapy was comparable between the groups (55.6% vs. 43.6%). In both groups, the most common reason for changing antifungal therapy was inadequate response to the treatment.

Among the isolates collected throughout the study period, 43 were revived and analyzed for antifungal susceptibility using standardized methods. The susceptibility results are presented in Table 5. Among *C. krusei* isolates, one was resistant to amphotericin B and another to posaconazole (NWT). One *C. tropicalis* isolate exhibited a posaconazole minimum inhibitory concentration above the epidemiologic cutoff (NWT). While there were no resistant *C. albicans* isolates, fluconazole resistance was detected in one *C. glabrata* isolate (25%) and two *C. parapsilosis* isolates (25%). These resistant *C. glabrata* and *C. parapsilosis* isolates were also cross-resistant to voriconazole with reduced susceptibility, but no cross-resistance to posaconazole was found.

### 3.4. Survival Outcomes

**Figure 4:**
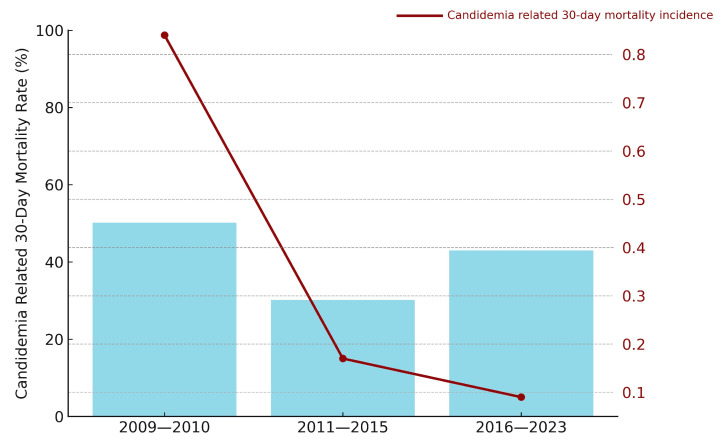
Temporal patterns of candidemia outcomes in AML patients.

The 30-day candidemia-related mortality rate was 40.7% among patients with AML compared to 61.5% in the non-AML patients (*p* = 0.096). In patients with AML, 30-day mortality rates and candidemia-related death rates per 1000 hospital-days, stratified by antifungal prophylaxis period, are shown together in Figure 4. There was no statistically significant difference in 30-day mortality rates according to prophylaxis periods (50%, 30%, and 42.9%; *p* = 0.711). However, linear regression demonstrated a significant temporal decline in candidemia-related death rate per 1000 AML hospital-days (β = −0.048; 95% CI, −0.080 to −0.016; *p* = 0.007; R^2^ = 0.446). Analysis of the data revealed a significant decrease in the death rate due to candidemia between the periods of 2009–2010 and 2016–2023, with rates declining from 0.84 to 0.09 deaths per 1000 hospital-days (*p* = 0.019). Additionally, the decrease in candidemia-related death rates from 0.84 to 0.17 per 1000 hospital-days between 2009–2010 and 2011–2015 was near the threshold of statistical significance (*p* = 0.051).

Binary logistic regression analyses of factors influencing 30-day candidemia-related mortality in AML patients and the entire cohort are presented in Table 6. Multivariate analysis revealed that *C. tropicalis*-associated candidemia was an independent risk factor for 30-day mortality (OR:18, *p* = 0.016). In the whole cohort, *C. tropicalis* and TPN administration were determined as independent risk factors that significantly increased the risk of 30-day mortality (OR 4.3 and OR 4.6, respectively; *p* = 0.045 and *p* = 0.015). However, antifungal prophylaxis was found to be a protective factor associated with a significant reduction in mortality risk (OR: 0.07; *p* = 0.017).

**Figure 5:**
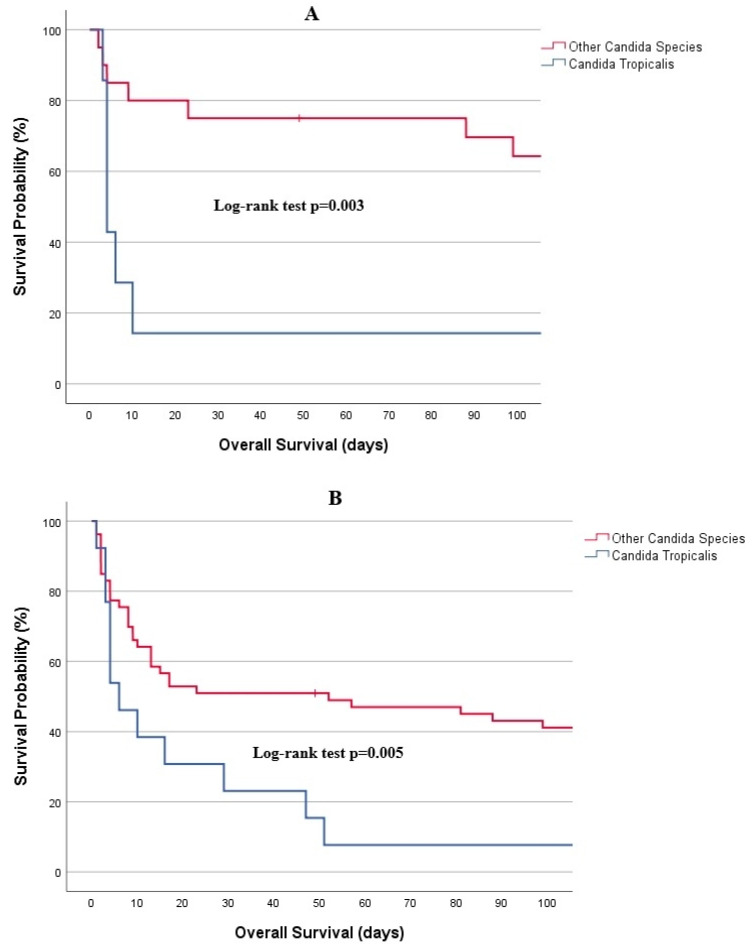
Kaplan–Meier analysis for 100-day survival, (**A**) AML patients, (**B**) overall cohort.

Survival curves by *Candida* species are depicted in Figure 5. Kaplan–Meier analysis demonstrated that individuals infected with *C. tropicalis* exhibited significantly lower survival rates at both 7 days and 30 days compared to those infected with other species. In the AML subgroup, the 7-day survival rate for *C. tropicalis* was 28.6%, and the 30-day rate was 14%, while the corresponding rates for other species were 85% and 75%, respectively (*p* = 0.003). In the overall cohort, the 7-day survival rate for *C. tropicalis* was 46.2%, and the 30-day rate was 23.1%, compared to 75.5% and 50.9% for other species (*p* = 0.005).

## 4. Discussion

Although international guidelines inform candidemia management, geographic variation in *Candida* species distribution and center-specific prophylaxis practices necessitate individualized treatment strategies [25,26,27]. In patients with AML, recent changes in antifungal prophylaxis have markedly impacted both candidemia incidence and causative species [28]. Nonetheless, persistently high mortality rates underscore the necessity to reevaluate current approaches [13]. In this context, obtaining and evaluating local epidemiological data is essential to optimize therapeutic decision-making. Over our 14 year observation, the adoption of routine posaconazole prophylaxis in patients with AML was associated with significant reductions in both candidemia incidence and candidemia-related mortality risk. While rates of *C. albicans* and potentially fatal *C. tropicalis* infections declined with posaconazole use, the frequency of *C. krusei* and other NAC species remained unchanged.

In studies conducted by the SEIFEM group comparing the incidence of candidemia in patients with hematological malignancies between 1999–2003 and 2011–2015, a significant reduction in candidemia—particularly among patients with AML receiving routine posaconazole prophylaxis—was demonstrated (4.8% vs. 1.5%) [29]. Posaconazole stands out for its efficacy against *C. krusei*, which is intrinsically resistant to fluconazole, and for its low cross-resistance rates against *C. tropicalis* and *C. parapsilosis*, species that may acquire fluconazole resistance [30,31,32]. In addition, among azoles, posaconazole has exhibited the highest overall efficacy against *Candida* species (93.7%) [33]. Clinical studies investigating the efficacy of this agent have repeatedly emphasized the critical role of adequate plasma concentrations in achieving therapeutic success. In particular, the suspension formulation is prone to variable absorption and pharmacokinetic interactions (e.g., with proton pump inhibitors), which may result in sub-therapeutic plasma levels [34]. In contrast, the extended-release tablet formulation achieves higher oral bioavailability and attains plasma levels comparable to those in intravenous administration [35]. Although a downward trend in candidemia incidence among AML patients was observed beginning in 2011, statistical significance was achieved only in the 2016–2023 period, corresponding to the introduction of extended-release posaconazole tablets at our institution. This finding suggests that overcoming the absorption-related limitations of the suspension formulation—thereby ensuring more stable plasma concentrations—may be more effective in preventing candidemia.

NAC species have been isolated with increasing frequency in BrC cases over time. *C. tropicalis, C. krusei*, *C. glabrata*, and *C. parapsilosis* are the most frequently reported pathogens [9,36]. Epidemiological data from Turkey likewise highlight *C. parapsilosis* and *C. tropicalis* as the predominant NAC species [3,37,38]. In this context, there is a significant increase in *C. tropicalis* and *C. krusei* infections in patients with hematological malignancies [39,40]. While the natural resistance to fluconazole is considered to play a role in the spread *of C. krusei* [32,40], the increase in *C. tropicalis* incidence is attributed to virulence factors such as suppression of cellular immunity as a result of neutropenia and susceptibility to biofilm formation following mucosal barrier damage [40]. In addition, extensive azole exposure may select for acquired resistance in *C. tropicalis*, contributing to its predominance [36]. Previous studies indicate that candidemia from these species has higher mortality rates compared to other *Candida* infections [41,42]. Candidemia, particularly cases linked to *C. tropicalis*, greatly decreases the 30-day survival rates [41,43,44]. In the present study, *C. krusei* and *C. tropicalis* were the most frequently isolated strains both in the AML subgroup and in the overall cohort. Consistent with the literature, *C. tropicalis*-associated candidemia cases had significantly lower 7- and 30-day survival rates, and this species was also found to be an independent risk factor for 30-day mortality.

Despite reported temporal declines in candidemia incidence, candidemia-related mortality rates have often remained unchanged [45]. Studies of mortality predictors have assessed microbiological variables such as type of candidemia, species of *Candida* [41,42], antifungal agent used in first-line therapy [36], as well as host-related factors, such as the presence of CVC, TPN administration [38], profound neutropenia, septic shock [36], and the requirement for mechanical ventilation [46]. Current evidence suggests that host-related factors may exert a more decisive influence on prognosis [47]. In a study evaluating risk factors for candidemia among allogeneic transplant recipients, age over 40 years and male sex were identified as independent risk factors for the development of candidemia, whereas no significant impact on prognosis was observed in the analysis of mortality [48]. In our cohort, we did not observe significant differences in candidemia incidence or outcomes based on age or sex. Consistent with these findings, we observed no significant changes in mortality over time among patients with AML with candidemia; instead, TPN administration and de novo candidemia emerged as independent predictors of 30-day mortality. Nevertheless, posaconazole prophylaxis was associated with a marked reduction in candidemia-related mortality rates per hospital-day.

There is no consensus among reports regarding the early initiation of echinocandins and its impact on prognosis. Although some studies suggest that early use may improve survival in critically ill patients, multivariable analyses have not definitively confirmed this benefit. However, it has been reported that L-AmB may be superior to echinocandins in *C. tropicalis*-associated candidemia [49,50]. In the present study, L-AmB was observed to be the most frequently selected agent for first-line empirical therapy, whereas echinocandin use was less common. Nevertheless, we did not observe an independent association between choice of antifungal agent and 30-day mortality.

Although *C. albicans* isolates generally remain azole-susceptible, increasing azole resistance among NAC species has been reported in recent years [10,51]. Elevated azole resistance in species such as *C. glabrata* and *C. parapsilosis* limits therapeutic alternatives and complicates clinical management [9,52]. Although echinocandins and L-AmB are commonly used in the treatment of these resistant infections, limited cases of resistance to these agents have also been reported, with resistance rates varying across different geographic regions [37,53]. As posaconazole does not easily form a uniform suspension in vitro, the accuracy of standard susceptibility testing for this drug is debated. In the present study, one *C. krusei* and one *C. tropicalis* isolate were classified as NWT for posaconazole; however, in the absence of molecular resistance testing, these findings warrant confirmation. We plan to investigate resistance genes in these isolates in the future. Fluconazole resistance detected in *C. parapsilosis* parallels the rates reported in Turkey and Mediterranean countries [52,54,55,56,57]. In a fluconazole-resistant *C. parapsilosis* isolate, voriconazole was also found as SDD/I and cross-resistance was demonstrated. Similarly, one *C. glabrata* isolate exhibited cross-resistance between fluconazole and voriconazole. Importantly, no cross-resistance to posaconazole was observed in fluconazole-resistant strains, suggesting a potential advantage for its prophylactic use. These findings underscore the necessity of ongoing surveillance of local resistance patterns and integration of antifungal susceptibility testing into clinical decision-making.

This study has several limitations. First, its retrospective, single-center design may limit generalizability despite ensuring consistency of data. Second, not all isolates underwent antifungal susceptibility testing, which may have resulted in underestimation of resistance patterns. Third, molecular analyses of azole resistance mechanisms were not performed; thus, the genetic factors underlying resistance remain uncharacterized. Finally, pharmacokinetic measurements (e.g., posaconazole plasma levels) were not included, which is another constraint in interpreting prophylactic efficacy.

## 5. Conclusions

The routine implementation of posaconazole prophylaxis in patients with AML was associated with a significant reduction in both candidemia incidence and candidemia-related deaths per 1000 AML hospital-days. This improvement was especially pronounced following the adoption of the extended-release tablet formulation. However, despite these positive developments, 30-day mortality rates in patients with candidemia remained high. *C. tropicalis* infections were associated with particularly poor short-term survival. These findings demonstrate that posaconazole-based prophylaxis effectively reduces candidemia burden in patients with AML, yet candidemia-related mortality remains a serious challenge. This highlights the necessity for individualized antifungal strategies and rapid diagnostic–therapeutic algorithms in high-risk populations. Future multicenter, prospective studies and molecular investigations into resistance mechanisms will contribute to the development of more effective candidemia management strategies.

## Figures and Tables

**Table 1:** General patient characteristics.

Variables	*n*: 66
**Median Age, years (IQR)**	48.5 (32–58)
**Sex, male (%)**	38 (57.6)
**Underlying Disease**	
*Acute Myeloid Leukemia*, (%)	27 (40.9)
*Acute Lymphoblastic Leukemia*, (%)	20 (30.3)
*Non-Hodgkin Lymphoma*, (%)	14 (21.2)
*Multiple Myeloma*, (%)	4 (6.1)
*Hodgkin Lymphoma*, (%)	1 (1.5)
**Disease Status**	
*Newly Diagnosed*, (%)	20 (30.3)
*Consolidation Phase*, (%)	8 (12.1)
*Relapsed/Refractory*, (%)	38 (57.6)
**Predisposing Factors**	
*Diabetes Mellitus*, (%)	8 (13.6)
*Systemic Corticosteroids*, (%)	43 (65.2)
*Central Venous Catheter*, (%)	34 (51.5)
*Total Parenteral Nutrition*, (%)	24 (36.4)
*Broad-spectrum Antimicrobial Agents*, (%)	63 (95.4)
*Hospital Stay (previous 90 days)*, (%)	30 (45.5)
*Neutropenia/ Severe Neutropenia*, (%)	55/49 (83.3/74.2)
**Candidemia Type**	
*Breakthrough Candidemia*, (%)	54 (81.8)
*De Novo Infection*, (%)	12 (18.2)
**Death Within 30 Days After Candidemia Onset, (%)**	35 (53)

IQR: interquartile range.

**Table 2:** *Candida* species distribution.

	AML Patients *n*: 27	Non-AML Patients *n*: 39	*p*-Value
***Candida albicans*, (%)**	3 (11.2)	6 (15.4)	0.946 ^b^
**Non-albicans *Candida,* (%)**	24 (88.8)	33 (84.6)	
*Candida krusei*, (%)	7 (25.9)	14 (42.4)	
*Candida tropicalis*, (%)	7 (25.9)	6 (18.1)	
*Candida parapsilosis*, (%)	5 (18.5)	5 (15.1)	
*Candida norvegensis/Candida inconspicua*, (%)	2 (7.4)	2 (6.1)	
*Candida glabrata*, (%)	1 (3.7)	2 (6.1)	
*Candida kefyr*, (%)	1 (3.7)	2 (6.1)	
*Other Candida Species ^a^*, (%)	1 (3.7)	2 (6.1)	

^a^: Other *Candida* species include *Candida utilis* (1 isolate), *Candida guilliermondii* (2 isolates), ^b^: Fisher–Freeman–Halton test.

**Table 3:** Comparison of candidemia incidence across different antifungal prophylaxis eras in AML patients (per 1000 patient-days).

Period	Total Patient-Days in Hospital	Candidemia Incidence (Median, IQR)	*p*-Value
2009–2010	5924	1.67 (1.52–1.81)	**0.022 ^a^**
2011–2015	17,336	0.48 (0.29–0.87)	
2016–2023	33,074	0.25 (0–0.35)	

AML: acute myeloid leukemia, IQR: interquartile range, ^a^: Kruskal–Wallis test, Bold value: Statistically significant.

**Table 4:** Overview of antifungal management and outcomes in AML patients and the overall candidemia cohort.

Clinical Factors	AML Patients *n*: 27	Non-AML Patients *n*: 39	*p*-Value
**Antifungal Prophylaxis, (%)**	27 (100)	27 (69.2)	**<0.001 ^a^**
*Fluconazole*, (%)	18 (66.7)	22 (81.5)	**0.002 ^a^**
*Posaconazole*, (%)	8 (29.6)	-	
*Itraconazole*, (%)	-	3 (11.1)	
*Liposomal Amphotericin B*, (%)	1 (3.7)	2 (7.4)	
**Initial Antifungal Treatment, (%)**			
*Fluconazole*, (%)	8 (29.6)	10 (25.6)	0.529 ^a^
*Liposomal Amphotericin B*, (%)	14 (51.9)	20 (51.3)	
*Echinocandin*, (%)	5 (18.5)	7 (17.9)	
*Voriconazole*, (%)	-	2 (5.2)	
**Modification of Initial Antifungal Treatment, (%)**	15 (55.6)	17 (43.6)	>0.999 ^a^
Due to *Lack of Clinical Response*	12 (80)	14 (82.4)	
Due to *Adverse Event*	3 (20)	3 (17.6)	
**Crude 30-day Mortality**	11 (40.7)	24 (61.5)	0.096 ^a^

^a^: Chi-squared test, Bold values: Statistically significant.

**Table 5:** Antifungal susceptibility results of clinical *Candida* isolates by species.

Species (No. of Tested)	Antifungal Agent	Resistant *n* (%)	SDD/I *n* (%)	Non-Wild-Type *n* (%)
***C. krusei* (20)**	Amphotericin B	-	-	1 (5)
	Itraconazole	-	-	-
	Posaconazole	-	-	1 (5)
	Voriconazole	-	-	-
	Anidulafungin	-	-	-
***C. tropicalis* (8)**	Amphotericin B	-	-	-
	Fluconazole	-	-	-
	Itraconazole	-	-	-
	Posaconazole	-	-	1 (12.5)
	Voriconazole	-	-	-
	Anidulafungin	-	-	-
***C. parapsilosis* (8)**	Amphotericin B	-	-	1 (12.5)
	Fluconazole	-	-	2 (25)
	Itraconazole	-	-	-
	Posaconazole	-	-	-
	Voriconazole	-	1 (12.5)	-
	Anidulafungin	-	-	-
***C. glabrata* (4)**	Amphotericin B	-	-	-
	Fluconazole	1 (25)	3 (75)	-
	Itraconazole	-	-	-
	Posaconazole	-	-	-
	Voriconazole	-	-	1 (25)
	Anidulafungin	-	-	-
***C. albicans* (3)**	Amphotericin B	-	-	-
	Fluconazole	-	-	-
	Itraconazole	-	-	-
	Posaconazole	-	-	-
	Voriconazole	-	-	-
	Anidulafungin	-	-	-

SDD: susceptible dose-dependent, I: intermediate.

**Table 6:** Logistic regression analysis of risk factors associated with 30-day crude mortality in AML patients with candidemia.

Variables		AML Patients Univariate Analysis (OR, 95% CI), *p*-Value	AML Patients Multivariate Analysis (OR, 95% CI), *p*-Value	Overall Cohort Univariate Analysis (OR, 95% CI), *p*-Value	Overall Cohort Multivariate Analysis (OR, 95% CI), *p*-Value
Age, (years)		1.006 (0.948–1.068) 0.845		1.003 (0.970–1.037) 0.873	
Gender	*Male (R)* vs. *Female*	0.625 (0.118–3.316) 0.581		1.038 (0.390–2.762) 0.940	
Infection Period	*<A (R)* vs. *≥B,C*	0.823 (0.308–2.204) 0.699		1.386 (0.731–2.630) 0.318	
Disease Status	*Active (R)* vs. *Remission*	0.301 (0.072–1.256) 0.100		0.660 (0.300–1.452) 0.302	
Diabetes Mellitus	*Absent (R)* vs. *Presence*	5.625 (0.500–63.282) 0.162		1.931 (0.440–8.483) 0.383	
Systemic Corticosteroids	*Presence (R)* vs. *Absent*	0.500 (0.105–2.379) 0.384		0.614 (0.219–1.717) 0.352	
Central Venous Catheter	*Presence (R)* vs. *Absent*	0.343 (0.070–1.684) 0.187		0.778 (0.295–2.051) 0.611	
Total Parenteral Nutrition	*Absent (R)* vs. *Presence*	2.500 (0.485–12.886) 0.273		4.412 (1.453–13.394) **0.009**	4.615 (1.342–15.869) **0.015**
Hospital Stay (previous 90 days)	*Absent (R)* vs. *Presence*	0.370 (0.059–2.323) 0.289		1.023 (0.387–2.700) 0.964	
Severe Neutropenia	*Absent (R)* vs. *Presence*	-		1.381 (0.457–4.174) 0.568	
Candidemia Type	*Denovo (R)* vs. *BrC*	-		0.073 (0.009–0.604) **0.015**	0.070 (0.008–0.628) **0.017**
Antifungal Prophylaxis	*Posa (R)* vs. *Flu*	2.095 (0.356–12.322) 0.413		2.222 (0.436–11.320) 0.336	
*Candida* Species	*Others (R)* vs. *Tropicalis*	18 (1.723–188.082) **0.016**	18 (1.723–188.082) **0.016**	2.337 (0.640–8.531) 0.199	4.301 (1.030–17.955) **0.045**
First Line Antifungal Therapy	*Others (R)* vs. *ECH*	0.300 (0.029–3.135) 0.315		0.371 (0.100–1.383) 0.140	

AML: acute myeloid leukemia, OR: odds ratio, CI: confidential interval, (R): reference category, A: 2009–2010, B: 2011–2015, C: 2016–2023, BrC: breakthrough candidemia, Posa: posaconazole, Flu: fluconazole, ECH: echinocandin, Bold values: statistically significant.

## Data Availability

The raw data supporting the conclusions of this article will be made available by the authors on request.

## References

[1] Denning D.W. (2024). Global Incidence and Mortality of Severe Fungal Disease. Lancet Infect. Dis..

[2] Cornely O.A., Gachot B., Akan H., Bassetti M., Uzun O., Kibbler C., Marchetti O., De Burghgraeve P., Ramadan S., Pylkkanen L. (2015). Epidemiology and Outcome of Fungemia in a Cancer Cohort of the Infectious Diseases Group (IDG) of the European Organization for Research and Treatment of Cancer (EORTC 65031). Clin. Infect. Dis..

[3] Şanlı K., Arslantaş E., Ceylan A.N., Öncel B., Özkorucu D., Özkan Karagenç A. (2024). Candidemia in Pediatric-Clinic: Frequency of Occurrence, Candida Species, Antifungal Susceptibilities, and Effects on Mortality (2020–2024). Diagnostics.

[4] Bongomin F., Gago S., Oladele R.O., Denning D.W. (2017). Global and Multi-National Prevalence of Fungal Diseases-Estimate Precision. J. Fungi.

[5] Tragiannidis A., Fegeler W., Rellensmann G., Debus V., Müller V., Hoernig-Franz I., Siam K., Pana Z.D., Jürgens H., Groll A.H. (2012). Candidaemia in a European Paediatric University Hospital: A 10-Year Observational Study. Clin. Microbiol. Infect..

[6] Arendrup M.C., Dzajic E., Jensen R.H., Johansen H.K., Kjældgaard P., Knudsen J.D., Kristensen L., Leitz C., Lemming L.E., Nielsen L. (2013). Epidemiological Changes with Potential Implication for Antifungal Prescription Recommendations for Fungaemia: Data from a Nationwide Fungaemia Surveillance Programme. Clin. Microbiol. Infect..

[7] Nucci M., Queiroz-Telles F., Alvarado-Matute T., Tiraboschi I.N., Cortes J., Zurita J., Guzman-Blanco M., Santolaya M.E., Thompson L., Sifuentes-Osornio J. (2013). Epidemiology of Candidemia in Latin America: A Laboratory-Based Survey. PLoS ONE.

[8] Pappas P.G., Kauffman C.A., Andes D.R., Clancy C.J., Marr K.A., Ostrosky-Zeichner L., Reboli A.C., Schuster M.G., Vazquez J.A., Walsh T.J. (2016). Clinical Practice Guideline for the Management of Candidiasis: 2016 Update by the Infectious Diseases Society of America. Clin. Infect. Dis..

[9] Posteraro B., De Carolis E., Criscuolo M., Ballanti S., De Angelis G., Del Principe M.I., Delia M., Fracchiolla N., Marchesi F., Nadali G. (2020). Candidaemia in Haematological Malignancy Patients from a SEIFEM Study: Epidemiological Patterns According to Antifungal Prophylaxis. Mycoses.

[10] Kothavade R.J., Kura M.M., Valand A.G., Panthaki M.H. (2010). Candida Tropicalis: Its Prevalence, Pathogenicity and Increasing Resistance to Fluconazole. J. Med. Microbiol..

[11] Malinovská Z., Čonková E., Váczi P. (2023). Biofilm Formation in Medically Important Candida Species. J. Fungi.

[12] Sanitá P.V., Zago C.E., De Oliveira Mima E.G., Pavarina A.C., Jorge J.H., MacHado A.L., Vergani C.E. (2014). In Vitro Evaluation of the Enzymatic Activity Profile of Non-Albicans Candida Species Isolated from Patients with Oral Candidiasis with or without Diabetes. Oral Surg. Oral Med. Oral Pathol. Oral Radiol..

[13] Sipsas N.V., Lewis R.E., Tarrand J., Hachem R., Rolston K.V., Raad I.I., Kontoyiannis D.P. (2009). Candidemia in Patients with Hematologic Malignancies in the Era of New Antifungal Agents (2001–2007): Stable Incidence but Changing Epidemiology of a Still Frequently Lethal Infection. Cancer.

[14] Cornely O.A., Maertens J., Winston D.J., Perfect J., Ullmann A.J., Walsh T.J., Helfgott D., Holowiecki J., Stockelberg D., Goh Y.-T. (2007). Posaconazole vs. Fluconazole or Itraconazole Prophylaxis in Patients with Neutropenia. N. Engl. J. Med..

[15] Pagano L., Maschmeyer G., Lamoth F., Blennow O., Xhaard A., Spadea M., Busca A., Cordonnier C., Maertens J., Guisado M.A. (2025). Primary Antifungal Prophylaxis in Hematological Malignancies. Updated Clinical Practice Guidelines by the European Conference on Infections in Leukemia (ECIL). Leukemia.

[16] Chau A.S., Mendrick C.A., Sabatelli F.J., Loebenberg D., McNicholas P.M. (2004). Application of Real-Time Quantitative PCR to Molecular Analysis of Candida Albicans Strains Exhibiting Reduced Susceptibility to Azoles. Antimicrob. Agents Chemother..

[17] Rodrigues C.F., Alves D.F., Henriques M. (2018). Combination of Posaconazole and Amphotericin B in the Treatment of Candida Glabrata Biofilms. Microorganisms.

[18] Kato H., Hagihara M., Shibata Y., Asai N., Yamagishi Y., Iwamoto T., Mikamo H. (2021). Comparison of Mortality between Echinocandins and Polyenes for an Initial Treatment of Candidemia: A Systematic Review and Meta-Analysis. J. Infect. Chemother..

[19] Antoniadou A., Torres H.A., Lewis R.E., Thornby J., Bodey G.P., Tarrand J.J., Han X.Y., Rolston K.V.I., Safdar A., Raad I.I. (2003). Candidemia in a Tertiary Care Cancer Center: In Vitro Susceptibility and Its Association with Outcome of Initial Antifungal Therapy. Medicine.

[20] Freifeld A.G., Bow E.J., Sepkowitz K.A., Boeckh M.J., Ito J.I., Mullen C.A., Raad I.I., Rolston K.V., Young J.A.H., Wingard J.R. (2011). Clinical Practice Guideline for the Use of Antimicrobial Agents in Neutropenic Patients with Cancer: 2010 Update by the Infectious Diseases Society of America. Clin. Infect. Dis..

[21] Hughes W.T., Armstrong D., Bodey G.P., Bow E.J., Brown A.E., Calandra T., Feld R., Pizzo P.A., Rolston K.V.I., Shenep J.L. (2002). 2002 Guidelines for the Use of Antimicrobial Agents in Neutropenic Patients with Cancer. Clin. Infect. Dis..

[22] (2017). Reference Method for Broth Dilution Antifungalsusceptibility Testing of Yeasts, 4th Edition.

[23] (2020). Reference Method for Broth Dilution Antifungalsusceptibility Testing of Yeasts, 2nd Edition.

[24] (2020). Reference Method for Broth Dilution Antifungalsusceptibility Testing of Yeasts, 3rd Edition.

[25] Pasqualotto A.C., Rosa D.D., Medeiros L.R., Severo L.C. (2006). Candidaemia and Cancer: Patients Are Not All the Same. BMC Infect. Dis..

[26] Gamaletsou M.N., Walsh T.J., Zaoutis T., Pagoni M., Kotsopoulou M., Voulgarelis M., Panayiotidis P., Vassilakopoulos T., Angelopoulou M.K., Marangos M. (2014). A Prospective, Cohort, Multicentre Study of Candidaemia in Hospitalized Adult Patients with Haematological Malignancies. Clin. Microbiol. Infect..

[27] McCort M.E., Tsai H. (2023). Epidemiology of Invasive Candidiasis in Patients with Hematologic Malignancy on Antifungal Prophylaxis. Mycopathologia.

[28] Pristov K.E., Ghannoum M.A. (2019). Resistance of Candida to Azoles and Echinocandins Worldwide. Clin. Microbiol. Infect..

[29] Pagano L., Dragonetti G., Cattaneo C., Marchesi F., Veggia B., Busca A., Candoni A., Prezioso L., Criscuolo M., Cesaro S. (2017). Changes in the Incidence of Candidemia and Related Mortality in Patients with Hematologic Malignancies in the Last Ten Years. A SEIFEM 2015-B Report. Haematologica.

[30] Mesini A., Mikulska M., Giacobbe D.R., Del Puente F., Gandolfo N., Codda G., Orsi A., Tassinari F., Beltramini S., Marchese A. (2020). Changing Epidemiology of Candidaemia: Increase in Fluconazole-Resistant Candida Parapsilosis. Mycoses.

[31] Pfaller M.A., Diekema D.J., Turnidge J.D., Castanheira M., Jones R.N. (2019). Twenty Years of the SENTRY Antifungal Surveillance Program: Results for Candida Species From 1997–2016. Open Forum Infect. Dis..

[32] Lindberg E., Hammarström H., Ataollahy N., Kondori N. (2019). Species Distribution and Antifungal Drug Susceptibilities of Yeasts Isolated from the Blood Samples of Patients with Candidemia. Sci. Rep..

[33] Maldonado N.A., Cano L.E., De Bedout C., Arbeláez C.A., Roncancio G., Tabares Á.M., Robledo C.G., Robledo J. (2014). Association of Clinical and Demographic Factors in Invasive Candidiasis Caused by Fluconazole-Resistant Candida Species: A Study in 15 Hospitals, Medellín, Colombia 2010–2011. Diagn. Microbiol. Infect. Dis..

[34] Desplanques P.Y., Burlacu R., Poinsignon V., Boussion H., Borget I., Wyplosz B., De Botton S., Billaud E., Chachaty E., Gachot B. (2014). Factors Influencing Posaconazole Plasmatic Concentrations in Patients Presenting with Acute Myeloid Leukemia. Médecine Mal. Infect..

[35] Patel T.S., Carver P.L., Eschenauer G.A. (2018). Are In Vitro Susceptibilities to Azole Antifungals Predictive of Clinical Outcome in the Treatment of Candidemia?. J. Clin. Microbiol..

[36] Ye N., Liu Z., Tang W., Li X., Chu W., Zhou Q. (2022). Systematic Characterization of Epidemiology, Antifungal Susceptibility, Risk Factors and Outcomes of Candidaemia: A Six-Year Chinese Study. Infect. Drug Resist..

[37] Yakut N., Kepenekli E., Ergenc Z., Baran E., Cerikcioglu N. (2021). Antifungal Susceptibility, Species Distribution and Risk Factors Associated with Mortality of Invasive Candidiasis in Children in Turkey: A Six-Year Retrospective, Single-Centre Study. J. Med. Mycol..

[38] Aydin S., Derin O., Sahin M., Dinleyici R., Yilmaz M., Ceylan B., Tosun A.I., Ozturk R., Mert A. (2022). Epidemiology of Nosocomial Candidemia, Mortality, and Antifungal Resistance: 7-Year Experience in Turkey. Jpn. J. Infect. Dis..

[39] Hachem R., Hanna H., Kontoyiannis D., Jiang Y., Raad I. (2008). The Changing Epidemiology of Invasive Candidiasis: Candida Glabrata and Candida Krusei as the Leading Causes of Candidemia in Hematologic Malignancy. Cancer.

[40] Chen X.C., Xu J., Wu D.P. (2020). Clinical Characteristics and Outcomes of Breakthrough Candidemia in 71 Hematologic Malignancy Patients and/or Allogeneic Hematopoietic Stem Cell Transplant Recipients: A Single-Center Retrospective Study From China, 2011–2018. Clin. Infect. Dis..

[41] Pongrácz J., Szabó T., Juhász E., Iván M., Kristóf K. (2025). Epidemiology, Risk Factors and Mortality of Fungal Bloodstream Infection: 14 Years of Experience at a Teaching Hospital. Mycopathologia.

[42] Pfaller M.A., Andes D.R., Diekema D.J., Horn D.L., Reboli A.C., Rotstein C., Franks B., Azie N.E. (2014). Epidemiology and Outcomes of Invasive Candidiasis Due to Non-Albicans Species of Candida in 2,496 Patients: Data from the Prospective Antifungal Therapy (PATH) Registry 2004–2008. PLoS ONE.

[43] Tan B.H., Chakrabarti A., Li R.Y., Patel A.K., Watcharananan S.P., Liu Z., Chindamporn A., Tan A.L., Sun P.L., Wu U.I. (2015). Incidence and Species Distribution of Candidaemia in Asia: A Laboratory-Based Surveillance Study. Clin. Microbiol. Infect..

[44] Puig-Asensio M., Ruiz-Camps I., Fernández-Ruiz M., Aguado J.M., Muñoz P., Valerio M., Delgado-Iribarren A., Merino P., Bereciartua E., Fortún J. (2015). Epidemiology and Outcome of Candidaemia in Patients with Oncological and Haematological Malignancies: Results from a Population-Based Surveillance in Spain. Clin. Microbiol. Infect..

[45] Kullberg B.J., Arendrup M.C. (2015). Invasive Candidiasis. N. Engl. J. Med..

[46] Abdullah N.M., Cheah S.K., Abdul Rahman R., Nor N.M., Maaya M., Musthafa Q.A. (2025). External Validation of Risk Prediction Score for Candidemia in Critically Ill Patients: A Retrospective Observational Study. J. Fungi.

[47] Schroeder M., Weber T., Denker T., Winterland S., Wichmann D., Rohde H., Ozga A.K., Fischer M., Kluge S. (2020). Epidemiology, Clinical Characteristics, and Outcome of Candidemia in Critically Ill Patients in Germany: A Single-Center Retrospective 10-Year Analysis. Ann. Intensive Care.

[48] Kimura S.I., Kameda K., Harada K., Saburi M., Okinaka K., Shinohara A., Uchida N., Nishijima A., Ozawa Y., Tanaka M. (2022). Risk and Predictive Factors for Candidemia After Allogeneic Hematopoietic Cell Transplantation: JSTCT Transplant Complications Working Group. Transplant. Cell. Ther..

[49] Bretagne S., Desnos-Ollivier M., Sitbon K., Lortholary O., Che D., Dromer F. (2021). No Impact of Fluconazole to Echinocandins Replacement as First-Line Therapy on the Epidemiology of Yeast Fungemia (Hospital-Driven Active Surveillance, 2004–2017, Paris, France). Front. Med..

[50] You L., Yao C., Yang F., Yang Q., Lan J., Song X., Shen J., Sheng X., Chen X., Tang H. (2020). Echinocandins versus Amphotericin B against Candida Tropicalis Fungemia in Adult Hematological Patients with Neutropenia: A Multicenter Retrospective Cohort Study. Infect. Drug Resist..

[51] Pfaller M.A., Jones R.N., Castanheira M. (2014). Regional Data Analysis of Candida Non-Albicans Strains Collected in United States Medical Sites over a 6-Year Period, 2006–2011. Mycoses.

[52] Semet C., Kazak E., Ener B., Ak S., Özkaya G., Ağca H., Heper Y., Yılmaz E., Akalın H. (2025). Risk Factors and Outcome for Bloodstream Infections Due to Fluconazole-Resistant Candida Parapsilosis: A 22-Year Single-Center Retrospective Study. Antimicrob. Resist. Infect. Control.

[53] Fuller J., Dingle T.C., Bull A., Shokoples S., Laverdière M., Baxter M.R., Adam H.J., Karlowsky J.A., Zhanel G.G. (2019). Species Distribution and Antifungal Susceptibility of Invasive Candida Isolates from Canadian Hospitals: Results of the CANWARD 2011–16 Study. J. Antimicrob. Chemother..

[54] Arikan-Akdagli S., Gülmez D., Doğan Ö., Çerikçioğlu N., Doluca Dereli M., Birinci A., Yıldıran Ş.T., Ener B., Öz Y., Metin D.Y. (2019). First Multicentre Report of in Vitro Resistance Rates in Candidaemia Isolates in Turkey. J. Glob. Antimicrob. Resist..

[55] Arendrup M.C., Arikan-Akdagli S., Jørgensen K.M., Barac A., Steinmann J., Toscano C., Arsenijevic V.A., Sartor A., Lass-Flörl C., Hamprecht A. (2023). European Candidaemia Is Characterised by Notable Differential Epidemiology and Susceptibility Pattern: Results from the ECMM Candida III Study. J. Infect..

[56] Kazak E., Akın H., Ener B., Sığırlı D., Özkan Ö., Gürcüoğlu E., Yılmaz E., Çelebi S., Akçağlar S., Akalın H. (2014). An Investigation of Candida Species Isolated from Blood Cultures during 17 Years in a University Hospital. Mycoses.

[57] Gürcüoğlu E., Ener B., Akalin H., Sinirtaş M., Evci C., Akçağlar S., Yilmaz E., Heper Y. (2010). Epidemiology of Nosocomial Candidaemia in a University Hospital: A 12-Year Study. Epidemiol. Infect..

